# 8-Hydr­oxy-2-methyl­quinolinium tetra­chlorido(quinolin-8-olato-κ^2^ *N*,*O*)stannate(IV) acetonitrile monosolvate

**DOI:** 10.1107/S160053681000810X

**Published:** 2010-03-06

**Authors:** Marzieh Vafaee, Gholamhossein Mohammadnezhad, Mostafa M. Amini, Seik Weng Ng

**Affiliations:** a Department of Chemistry, General Campus, Shahid Beheshti University, Tehran 1983963113, Iran; b Department of Chemistry, University of Malaya, 50603 Kuala Lumpur, Malaysia

## Abstract

In the title solvated salt, (C_10_H_10_NO)[SnCl_4_(C_9_H_6_NO)]·CH_3_CN, the Sn^IV^ atom is chelated by the *N*,*O*-bidentate 8-hydroxy­quinolinate ligand and four chloride ions, generating a distorted SnONCl_4_ octa­hedral coordination geometry for the metal. In the crystal, the cations are linked to the anions and the solvent mol­ecules by O—H⋯O and N—H⋯N hydrogen bonds, respectively.

## Related literature

For the spectroscopic characterization of the tetra­chlorido(quinolinato)stannate(IV) anion in other salts, see: Cunning­ham *et al.* (1977 ▶); Douek *et al.* (1967 ▶); Frazer & Goffer (1996 ▶); Frazer & Rimmer (1968 ▶); Greenwood & Ruddick (1967 ▶). For the structures of dichlorido­bis(quinolin-8-olato)tin and dichloridobis(2-methyl­quinolin-8-olato)tin, see: Archer *et al.* (1987 ▶); Lo & Ng (2009 ▶). For a related structure, see: Mohammadnezhad *et al.* (2010 ▶).
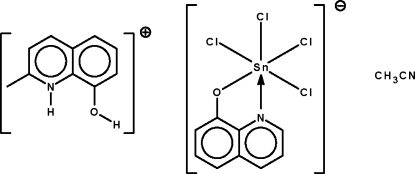


## Experimental

### 

#### Crystal data

(C_10_H_10_NO)[SnCl_4_(C_9_H_6_NO)]·C_2_H_3_N
*M* *_r_* = 605.88
Monoclinic, 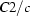

*a* = 29.1672 (14) Å
*b* = 10.9415 (5) Å
*c* = 15.4907 (8) Å
β = 99.9411 (6)°
*V* = 4869.4 (4) Å^3^
*Z* = 8
Mo *K*α radiation
μ = 1.51 mm^−1^
*T* = 296 K
0.40 × 0.30 × 0.20 mm

#### Data collection

Bruker SMART APEX diffractometer
Absorption correction: multi-scan (*SADABS*; Sheldrick, 1996 ▶) *T*_min_ = 0.583, *T*_max_ = 0.752
22804 measured reflections
5594 independent reflections
4663 reflections with *I* > 2σ(*I*)
*R*_int_ = 0.027

#### Refinement

*R*[*F* ^2^ > 2σ(*F* ^2^)] = 0.023
*wR*(*F* ^2^) = 0.064
*S* = 1.02
5594 reflections
290 parameters
2 restraints
H atoms treated by a mixture of independent and constrained refinement
Δρ_max_ = 0.28 e Å^−3^
Δρ_min_ = −0.54 e Å^−3^

### 

Data collection: *APEX2* (Bruker, 2009 ▶); cell refinement: *SAINT* (Bruker, 2009 ▶); data reduction: *SAINT*; program(s) used to solve structure: *SHELXS97* (Sheldrick, 2008 ▶); program(s) used to refine structure: *SHELXL97* (Sheldrick, 2008 ▶); molecular graphics: *X-SEED* (Barbour, 2001 ▶); software used to prepare material for publication: *publCIF* (Westrip, 2010 ▶).

## Supplementary Material

Crystal structure: contains datablocks global, I. DOI: 10.1107/S160053681000810X/hb5348sup1.cif

Structure factors: contains datablocks I. DOI: 10.1107/S160053681000810X/hb5348Isup2.hkl

Additional supplementary materials:  crystallographic information; 3D view; checkCIF report

## Figures and Tables

**Table:** 

Sn1—N1	2.203 (2)
Sn1—O1	2.077 (1)
Sn1—Cl1	2.3741 (6)
Sn1—Cl2	2.4055 (6)
Sn1—Cl3	2.4130 (6)
Sn1—Cl4	2.4176 (6)

**Table:** 

O1—Sn1—N1	78.08 (6)

**Table 2:** Hydrogen-bond geometry (Å, °)

*D*—H⋯*A*	*D*—H	H⋯*A*	*D*⋯*A*	*D*—H⋯*A*
O2—H2o⋯O1	0.85 (1)	1.91 (2)	2.715 (2)	158 (3)
N2—H2n⋯N3	0.85 (1)	2.15 (1)	2.972 (3)	162 (2)

**Fig. 1.**
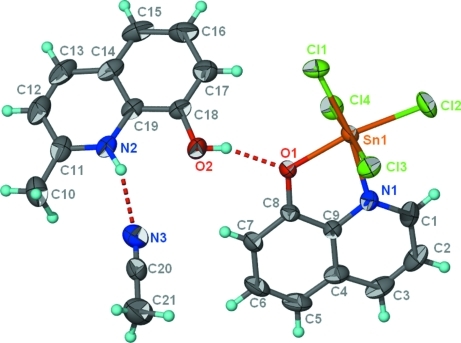
The molecular structure of (I): displacement ellipsoids are drawn at the 50% probability level and H atoms are of arbitrary radius.

## supplementary crystallographic information

### Comment

The 8-hydroxyquinoline anion furnishes a number of compounds with both inorganic tin(IV) and organotin(IV) systems; for example, the crystal structure of dichlorobis(quinolin-8-olato)tin has been known some time back (Archer *et al.*, 1987). The presence of a methyl substituent in the 2-position introduces steric problems (Mohammadnezhad *et al.*, 2010); this may affect the ability of the complexes to crystallize well so that fewer such complexes have been reported. The crystal structure of dichlorobis(2-methylquinolin-8-olato)tin has only recently been reported (Lo & Ng, 2009). On the other hand, mixed chelate complexes are difficult to synthesize as the compounds disproportionate into the symmetrical derivatives.

The reaction of tin(IV) chloride with 8-hydroxyquinoline and 2-methyl-8-hydroxyquinoline in acetonitrile yields instead the salt, 8-hydroxy-2-methylquinolinium tetrachloro(quinolin-8-olato)stannate as the acetonitrile solvate (Scheme I, Fig. 1). Owing to the steric bulk of the methyl group, the 2-methy-8-hydroxyquinoline component does not engage in binding to the tin atom but merely functions as a proton abstractor. In the salt, the tin atom is chelated by the 8-hydroxyquinolinato unit and it exists in an octahedral coordination geometry. The tin-chlorine bonds *trans* to the chelating atoms are significantly shorter than the other tin-chlorine bonds (Table 1). The cation is linked to the anion by an O–H···O hydrogen bond; the cation is linked to solvent molecule by an N–H···N hydrogen bond (Table 2). The stannate has been spectroscopically characterized in other salts (Cunningham *et al.*, 1977; Douek *et al., *1967; Frazer & Goffer, 1996; Frazer & Rimmer, 1968; Greenwood & Ruddick, 1967).

### Experimental

Stannic chloride pentahydrate(1 mmol, 0.35 g), 8-hydroxyquinoline (1 mmol, 0.15 g) and 2-methyl-8-hydroxyquinoline (1 mmol, 0.16 g) were placed in a convection tube; the tube was filled with acetonitrile and kept at 333 K. Yellow prisms of (I) were collected after a week.

### Refinement

Carbon-bound H-atoms were placed in calculated positions (C—H 0.93 to 0.96 Å) and were included in the refinement in the riding model approximation, with *U*(H) set to 1.2 to 1.5*U*(C). The nitrogen- and oxygen-bound ones were located in a difference Fourier map, and were refined with distance restraints of N–H = O–H 0.86±0.01 Å; their temperature factors were refined.

### Crystal data

**Table:**

(C_10_H_10_NO)[SnCl_4_(C_9_H_6_NO)]·C_2_H_3_N	*F*(000) = 2400
*M**_r_* = 605.88	*D*_x_ = 1.653 Mg m^−^^3^
Monoclinic, *C*2/*c*	Melting point: 408 K
Hall symbol: -C 2yc	Mo *K*α radiation, λ = 0.71073 Å
*a* = 29.1672 (14) Å	Cell parameters from 9937 reflections
*b* = 10.9415 (5) Å	θ = 2.3–28.3°
*c* = 15.4907 (8) Å	µ = 1.51 mm^−^^1^
β = 99.9411 (6)°	*T* = 296 K
*V* = 4869.4 (4) Å^3^	Prism, yellow
*Z* = 8	0.40 × 0.30 × 0.20 mm

### Data collection

**Table:**

Bruker SMART APEX diffractometer	5594 independent reflections
Radiation source: fine-focus sealed tube	4663 reflections with *I* > 2σ(*I*)
graphite	*R*_int_ = 0.027
ω scans	θ_max_ = 27.5°, θ_min_ = 1.4°
Absorption correction: multi-scan (*SADABS*; Sheldrick, 1996)	*h* = −37→37
*T*_min_ = 0.583, *T*_max_ = 0.752	*k* = −14→14
22804 measured reflections	*l* = −20→20

### Refinement

**Table:**

Refinement on *F*^2^	Primary atom site location: structure-invariant direct methods
Least-squares matrix: full	Secondary atom site location: difference Fourier map
*R*[*F*^2^ > 2σ(*F*^2^)] = 0.023	Hydrogen site location: inferred from neighbouring sites
*wR*(*F*^2^) = 0.064	H atoms treated by a mixture of independent and constrained refinement
*S* = 1.02	*w* = 1/[σ^2^(*F*_o_^2^) + (0.0319*P*)^2^ + 3.2971*P*] where *P* = (*F*_o_^2^ + 2*F*_c_^2^)/3
5594 reflections	(Δ/σ)_max_ = 0.001
290 parameters	Δρ_max_ = 0.28 e Å^−^^3^
2 restraints	Δρ_min_ = −0.54 e Å^−^^3^

### Fractional atomic coordinates and isotropic or equivalent isotropic displacement parameters (Å^2^)

**Table:**

	*x*	*y*	*z*	*U*_iso_*/*U*_eq_	
Sn1	0.395979 (5)	0.698425 (12)	0.408891 (9)	0.03485 (6)	
Cl1	0.37060 (2)	0.76784 (6)	0.53800 (4)	0.05477 (15)	
Cl2	0.40349 (2)	0.89500 (5)	0.34322 (5)	0.06272 (18)	
Cl3	0.476603 (19)	0.70536 (6)	0.47849 (4)	0.04984 (14)	
Cl4	0.31714 (2)	0.68113 (6)	0.33007 (4)	0.05257 (15)	
O1	0.39266 (5)	0.51704 (12)	0.44682 (9)	0.0390 (3)	
O2	0.34376 (6)	0.38174 (17)	0.54651 (13)	0.0573 (5)	
H2O	0.3527 (11)	0.436 (2)	0.5137 (19)	0.092 (12)*	
N1	0.41915 (6)	0.60155 (16)	0.29942 (11)	0.0395 (4)	
N2	0.29662 (7)	0.20670 (18)	0.61441 (12)	0.0443 (4)	
H2N	0.3234 (5)	0.197 (2)	0.6006 (16)	0.044 (7)*	
N3	0.38826 (10)	0.1151 (2)	0.58312 (19)	0.0753 (7)	
C1	0.43162 (9)	0.6467 (3)	0.22790 (15)	0.0533 (6)	
H1	0.4306	0.7308	0.2191	0.064*	
C2	0.44621 (9)	0.5712 (3)	0.16521 (16)	0.0621 (7)	
H2	0.4549	0.6050	0.1154	0.075*	
C3	0.44759 (9)	0.4489 (3)	0.17715 (16)	0.0614 (7)	
H3	0.4571	0.3987	0.1351	0.074*	
C4	0.43490 (8)	0.3968 (2)	0.25239 (15)	0.0488 (6)	
C5	0.43577 (10)	0.2710 (3)	0.2723 (2)	0.0648 (8)	
H5	0.4447	0.2149	0.2332	0.078*	
C6	0.42365 (10)	0.2317 (2)	0.3480 (2)	0.0618 (7)	
H6	0.4248	0.1484	0.3603	0.074*	
C7	0.40938 (8)	0.31228 (19)	0.40858 (17)	0.0453 (5)	
H7	0.4015	0.2821	0.4602	0.054*	
C8	0.40704 (7)	0.43536 (18)	0.39199 (14)	0.0358 (4)	
C9	0.42052 (7)	0.47834 (19)	0.31345 (13)	0.0364 (4)	
C10	0.30128 (11)	−0.0040 (3)	0.6638 (2)	0.0714 (8)	
H10A	0.3128	−0.0270	0.6117	0.107*	
H10B	0.2803	−0.0657	0.6777	0.107*	
H10C	0.3269	0.0042	0.7116	0.107*	
C11	0.27629 (9)	0.1142 (2)	0.64902 (15)	0.0547 (6)	
C12	0.23234 (10)	0.1348 (3)	0.67019 (19)	0.0693 (8)	
H12	0.2173	0.0719	0.6946	0.083*	
C13	0.21137 (9)	0.2439 (3)	0.65597 (19)	0.0687 (8)	
H13	0.1821	0.2549	0.6707	0.082*	
C14	0.23274 (8)	0.3424 (3)	0.61921 (15)	0.0530 (6)	
C15	0.21283 (10)	0.4587 (3)	0.6017 (2)	0.0702 (8)	
H15	0.1831	0.4746	0.6129	0.084*	
C16	0.23708 (11)	0.5477 (3)	0.5685 (2)	0.0718 (8)	
H16	0.2240	0.6251	0.5588	0.086*	
C17	0.28137 (10)	0.5257 (3)	0.54830 (18)	0.0617 (7)	
H17	0.2971	0.5879	0.5247	0.074*	
C18	0.30146 (8)	0.4131 (2)	0.56320 (15)	0.0456 (5)	
C19	0.27715 (8)	0.3204 (2)	0.59882 (14)	0.0431 (5)	
C20	0.42250 (11)	0.0664 (2)	0.58977 (18)	0.0582 (7)	
C21	0.46668 (12)	0.0043 (3)	0.5990 (3)	0.0996 (13)	
H21A	0.4639	−0.0652	0.5606	0.149*	
H21B	0.4758	−0.0224	0.6585	0.149*	
H21C	0.4898	0.0592	0.5840	0.149*	

### Atomic displacement parameters (Å^2^)

**Table:**

	*U*^11^	*U*^22^	*U*^33^	*U*^12^	*U*^13^	*U*^23^
Sn1	0.03691 (9)	0.02715 (8)	0.04003 (8)	0.00082 (5)	0.00535 (6)	0.00091 (5)
Cl1	0.0529 (3)	0.0571 (4)	0.0555 (3)	0.0079 (3)	0.0127 (3)	−0.0166 (3)
Cl2	0.0668 (4)	0.0322 (3)	0.0873 (5)	−0.0024 (3)	0.0084 (3)	0.0155 (3)
Cl3	0.0356 (3)	0.0614 (4)	0.0513 (3)	0.0017 (2)	0.0039 (2)	−0.0024 (3)
Cl4	0.0404 (3)	0.0569 (4)	0.0562 (3)	−0.0032 (2)	−0.0035 (2)	0.0031 (3)
O1	0.0508 (9)	0.0289 (7)	0.0405 (8)	0.0029 (6)	0.0172 (6)	0.0023 (6)
O2	0.0440 (9)	0.0543 (11)	0.0803 (13)	0.0083 (8)	0.0295 (9)	0.0185 (9)
N1	0.0414 (10)	0.0411 (10)	0.0362 (9)	−0.0020 (8)	0.0074 (7)	0.0025 (7)
N2	0.0389 (10)	0.0557 (12)	0.0395 (9)	−0.0072 (9)	0.0099 (8)	−0.0046 (8)
N3	0.0781 (18)	0.0534 (14)	0.099 (2)	0.0056 (13)	0.0267 (15)	0.0007 (13)
C1	0.0574 (15)	0.0613 (16)	0.0421 (12)	−0.0056 (12)	0.0111 (11)	0.0116 (11)
C2	0.0541 (15)	0.099 (2)	0.0353 (12)	−0.0032 (15)	0.0130 (11)	0.0036 (13)
C3	0.0488 (14)	0.095 (2)	0.0413 (13)	0.0014 (14)	0.0098 (11)	−0.0218 (14)
C4	0.0411 (12)	0.0569 (15)	0.0481 (13)	0.0005 (11)	0.0069 (10)	−0.0171 (11)
C5	0.0618 (17)	0.0542 (16)	0.0785 (19)	0.0066 (13)	0.0119 (14)	−0.0315 (14)
C6	0.0592 (16)	0.0318 (12)	0.093 (2)	0.0027 (11)	0.0107 (15)	−0.0111 (13)
C7	0.0413 (12)	0.0335 (11)	0.0608 (14)	0.0005 (9)	0.0079 (10)	0.0024 (10)
C8	0.0314 (10)	0.0326 (10)	0.0429 (11)	0.0017 (8)	0.0049 (8)	−0.0023 (8)
C9	0.0305 (10)	0.0399 (11)	0.0382 (10)	−0.0002 (8)	0.0038 (8)	−0.0053 (8)
C10	0.084 (2)	0.0607 (18)	0.0682 (18)	−0.0182 (16)	0.0103 (15)	0.0090 (14)
C11	0.0583 (15)	0.0628 (16)	0.0432 (12)	−0.0222 (13)	0.0089 (11)	−0.0087 (11)
C12	0.0610 (17)	0.087 (2)	0.0643 (17)	−0.0347 (17)	0.0225 (14)	−0.0148 (16)
C13	0.0393 (14)	0.105 (2)	0.0656 (17)	−0.0219 (16)	0.0196 (12)	−0.0243 (17)
C14	0.0364 (12)	0.0810 (18)	0.0420 (12)	−0.0035 (12)	0.0073 (10)	−0.0158 (12)
C15	0.0425 (14)	0.103 (2)	0.0658 (17)	0.0164 (16)	0.0119 (13)	−0.0178 (17)
C16	0.0644 (18)	0.079 (2)	0.0728 (19)	0.0302 (16)	0.0144 (15)	−0.0023 (16)
C17	0.0585 (16)	0.0626 (17)	0.0656 (17)	0.0112 (13)	0.0152 (13)	0.0048 (13)
C18	0.0381 (11)	0.0555 (14)	0.0443 (12)	0.0036 (10)	0.0102 (9)	−0.0019 (10)
C19	0.0350 (11)	0.0592 (14)	0.0345 (10)	−0.0042 (10)	0.0044 (9)	−0.0085 (9)
C20	0.0721 (19)	0.0400 (13)	0.0665 (17)	−0.0085 (13)	0.0229 (14)	−0.0031 (12)
C21	0.068 (2)	0.070 (2)	0.167 (4)	−0.0003 (17)	0.036 (2)	−0.001 (2)

### Geometric parameters (Å, °)

**Table:**

Sn1—N1	2.203 (2)	C6—H6	0.9300
Sn1—O1	2.077 (1)	C7—C8	1.371 (3)
Sn1—Cl1	2.3741 (6)	C7—H7	0.9300
Sn1—Cl2	2.4055 (6)	C8—C9	1.422 (3)
Sn1—Cl3	2.4130 (6)	C10—C11	1.482 (4)
Sn1—Cl4	2.4176 (6)	C10—H10A	0.9600
O1—C8	1.349 (2)	C10—H10B	0.9600
O2—C18	1.348 (3)	C10—H10C	0.9600
O2—H2O	0.849 (10)	C11—C12	1.395 (4)
N1—C1	1.320 (3)	C12—C13	1.341 (5)
N1—C9	1.365 (3)	C12—H12	0.9300
N2—C11	1.332 (3)	C13—C14	1.413 (4)
N2—C19	1.371 (3)	C13—H13	0.9300
N2—H2N	0.852 (10)	C14—C19	1.406 (3)
N3—C20	1.121 (4)	C14—C15	1.406 (4)
C1—C2	1.396 (4)	C15—C16	1.356 (4)
C1—H1	0.9300	C15—H15	0.9300
C2—C3	1.351 (4)	C16—C17	1.401 (4)
C2—H2	0.9300	C16—H16	0.9300
C3—C4	1.403 (4)	C17—C18	1.367 (3)
C3—H3	0.9300	C17—H17	0.9300
C4—C5	1.410 (4)	C18—C19	1.404 (3)
C4—C9	1.415 (3)	C20—C21	1.442 (4)
C5—C6	1.352 (4)	C21—H21A	0.9600
C5—H5	0.9300	C21—H21B	0.9600
C6—C7	1.402 (4)	C21—H21C	0.9600
			
O1—Sn1—N1	78.08 (6)	O1—C8—C9	118.80 (18)
O1—Sn1—Cl1	91.77 (4)	C7—C8—C9	118.2 (2)
N1—Sn1—Cl1	169.83 (5)	N1—C9—C4	121.3 (2)
O1—Sn1—Cl2	170.48 (4)	N1—C9—C8	117.27 (18)
N1—Sn1—Cl2	92.42 (5)	C4—C9—C8	121.4 (2)
Cl1—Sn1—Cl2	97.74 (3)	C11—C10—H10A	109.5
O1—Sn1—Cl3	89.60 (4)	C11—C10—H10B	109.5
N1—Sn1—Cl3	87.37 (5)	H10A—C10—H10B	109.5
Cl1—Sn1—Cl3	92.01 (2)	C11—C10—H10C	109.5
Cl2—Sn1—Cl3	90.45 (2)	H10A—C10—H10C	109.5
O1—Sn1—Cl4	88.93 (4)	H10B—C10—H10C	109.5
N1—Sn1—Cl4	88.00 (5)	N2—C11—C12	117.6 (3)
Cl1—Sn1—Cl4	92.45 (2)	N2—C11—C10	118.9 (2)
Cl2—Sn1—Cl4	90.26 (2)	C12—C11—C10	123.5 (3)
Cl3—Sn1—Cl4	175.34 (2)	C13—C12—C11	121.1 (3)
C8—O1—Sn1	114.85 (12)	C13—C12—H12	119.4
C18—O2—H2O	109 (2)	C11—C12—H12	119.4
C1—N1—C9	119.9 (2)	C12—C13—C14	121.6 (3)
C1—N1—Sn1	129.17 (17)	C12—C13—H13	119.2
C9—N1—Sn1	110.92 (13)	C14—C13—H13	119.2
C11—N2—C19	124.1 (2)	C19—C14—C15	118.5 (3)
C11—N2—H2N	119.6 (16)	C19—C14—C13	116.6 (3)
C19—N2—H2N	116.3 (16)	C15—C14—C13	124.9 (3)
N1—C1—C2	121.6 (3)	C16—C15—C14	119.9 (3)
N1—C1—H1	119.2	C16—C15—H15	120.1
C2—C1—H1	119.2	C14—C15—H15	120.1
C3—C2—C1	119.8 (2)	C15—C16—C17	121.5 (3)
C3—C2—H2	120.1	C15—C16—H16	119.2
C1—C2—H2	120.1	C17—C16—H16	119.2
C2—C3—C4	120.7 (2)	C18—C17—C16	120.1 (3)
C2—C3—H3	119.7	C18—C17—H17	119.9
C4—C3—H3	119.7	C16—C17—H17	119.9
C3—C4—C5	125.5 (2)	O2—C18—C17	125.2 (2)
C3—C4—C9	116.8 (2)	O2—C18—C19	115.6 (2)
C5—C4—C9	117.7 (2)	C17—C18—C19	119.1 (2)
C6—C5—C4	120.2 (2)	N2—C19—C18	120.2 (2)
C6—C5—H5	119.9	N2—C19—C14	119.0 (2)
C4—C5—H5	119.9	C18—C19—C14	120.8 (2)
C5—C6—C7	122.2 (2)	N3—C20—C21	179.5 (4)
C5—C6—H6	118.9	C20—C21—H21A	109.5
C7—C6—H6	118.9	C20—C21—H21B	109.5
C8—C7—C6	120.2 (2)	H21A—C21—H21B	109.5
C8—C7—H7	119.9	C20—C21—H21C	109.5
C6—C7—H7	119.9	H21A—C21—H21C	109.5
O1—C8—C7	122.9 (2)	H21B—C21—H21C	109.5
			
N1—Sn1—O1—C8	2.45 (13)	C3—C4—C9—N1	0.0 (3)
Cl1—Sn1—O1—C8	−176.95 (13)	C5—C4—C9—N1	179.4 (2)
Cl3—Sn1—O1—C8	−84.95 (13)	C3—C4—C9—C8	−179.8 (2)
Cl4—Sn1—O1—C8	90.63 (13)	C5—C4—C9—C8	−0.5 (3)
O1—Sn1—N1—C1	179.6 (2)	O1—C8—C9—N1	1.6 (3)
Cl1—Sn1—N1—C1	−177.0 (2)	C7—C8—C9—N1	−178.21 (19)
Cl2—Sn1—N1—C1	0.1 (2)	O1—C8—C9—C4	−178.54 (18)
Cl3—Sn1—N1—C1	−90.3 (2)	C7—C8—C9—C4	1.6 (3)
Cl4—Sn1—N1—C1	90.2 (2)	C19—N2—C11—C12	0.3 (3)
O1—Sn1—N1—C9	−1.57 (13)	C19—N2—C11—C10	−179.2 (2)
Cl1—Sn1—N1—C9	1.8 (4)	N2—C11—C12—C13	0.1 (4)
Cl2—Sn1—N1—C9	178.93 (13)	C10—C11—C12—C13	179.6 (3)
Cl3—Sn1—N1—C9	88.58 (13)	C11—C12—C13—C14	−0.1 (4)
Cl4—Sn1—N1—C9	−90.90 (13)	C12—C13—C14—C19	−0.3 (4)
C9—N1—C1—C2	0.2 (3)	C12—C13—C14—C15	179.4 (3)
Sn1—N1—C1—C2	178.98 (17)	C19—C14—C15—C16	−2.0 (4)
N1—C1—C2—C3	0.2 (4)	C13—C14—C15—C16	178.2 (3)
C1—C2—C3—C4	−0.6 (4)	C14—C15—C16—C17	2.0 (5)
C2—C3—C4—C5	−178.9 (3)	C15—C16—C17—C18	−1.0 (5)
C2—C3—C4—C9	0.5 (4)	C16—C17—C18—O2	−179.9 (3)
C3—C4—C5—C6	178.6 (3)	C16—C17—C18—C19	0.0 (4)
C9—C4—C5—C6	−0.7 (4)	C11—N2—C19—C18	179.1 (2)
C4—C5—C6—C7	0.8 (4)	C11—N2—C19—C14	−0.8 (3)
C5—C6—C7—C8	0.4 (4)	O2—C18—C19—N2	0.0 (3)
Sn1—O1—C8—C7	176.79 (17)	C17—C18—C19—N2	−179.9 (2)
Sn1—O1—C8—C9	−3.1 (2)	O2—C18—C19—C14	179.8 (2)
C6—C7—C8—O1	178.6 (2)	C17—C18—C19—C14	−0.1 (3)
C6—C7—C8—C9	−1.6 (3)	C15—C14—C19—N2	−179.1 (2)
C1—N1—C9—C4	−0.3 (3)	C13—C14—C19—N2	0.7 (3)
Sn1—N1—C9—C4	−179.30 (16)	C15—C14—C19—C18	1.1 (3)
C1—N1—C9—C8	179.5 (2)	C13—C14—C19—C18	−179.1 (2)
Sn1—N1—C9—C8	0.5 (2)		

### Hydrogen-bond geometry (Å, °)

**Table:**

*D*—H···*A*	*D*—H	H···*A*	*D*···*A*	*D*—H···*A*
O2—H2o···O1	0.85 (1)	1.91 (2)	2.715 (2)	158 (3)
N2—H2n···N3	0.85 (1)	2.15 (1)	2.972 (3)	162 (2)
